# Differential expression of miRNAs and their targets in wax-deficient rapeseed

**DOI:** 10.1038/s41598-019-48439-z

**Published:** 2019-08-21

**Authors:** Tingting Liu, Jingquan Tang, Li Chen, Jiayue Zeng, Jing Wen, Bin Yi, Chaozhi Ma, Jinxing Tu, Tingdong Fu, Jinxiong Shen

**Affiliations:** National Key Laboratory of Crop Genetic Improvement, National Center of Rapeseed Improvement, Huazhong Agricultural University, Wuhan, Hubei China

**Keywords:** miRNAs, Plant morphogenesis

## Abstract

The cuticle of a plant, composed of cutin and wax, is the outermost hydrophobic layer covering the epidermis of all its aerial organs, protecting it from many abiotic and biotic stresses. The biosynthesis and regulation pathways of wax components have been well studied, whereas there are fewer reports on the small RNA-involved post-transcriptional regulation of wax biosynthesis in plants, particularly in *Brassica napus*. Previously, we conducted a study on a glossy mutant of rapeseed, and we assumed that there was a dominant repressor to inhibit the expression of wax-related genes. To verify this hypothesis and investigate the function of small RNAs in wax biosynthesis in *B. napus*, we constructed four small RNA libraries from the stem epidermis of wax-deficient mutant and wild-type plants for sequencing. Subsequently, 43,840,451 clean reads were generated and 24 nt sequences represented the dominant percentage. In total, 300 unique known miRNAs were identified and eight of them showed differential expression. In addition, the expression levels of six novel miRNAs were altered. Surprisingly, we found that four up-regulated miRNAs in the wax-deficient plants, bna-miR408b-5p, bna-miR165b-5p, bna-miR160a-3p, and bna-miR398-5p, were all complementary strands of their corresponding mature strands. Stem-loop qRT-PCR verified that the expression of bna-miR165a-5p was increased in the mutant stems, while its putative target, *BnaA06g40560D* (*CYP96A2*), was down-regulated. In addition, the expression of bna-miR827a was detected to be down-regulated in glossy mutant. 5′ RACE experimental data showed that bna-miR827a cleaves three *NITROGEN LIMITATION ADAPTATION (NLA)* genes (*BnaC08g45940D*, *BnaA10g01450D* and *BnaC05g01480D*). The down-regulation of bna-miR827a resulted in decreased cleavage on its targets, and led to the up-regulation of its targets, especially *BnaA10g01450D* gene. These results showed that bna-miR165a-5p might participate in wax biosynthesis process by regulating its putative target *BnaA06g40560D* (*CYP96A2*). The expression levels of a phosphate (Pi)-related miRNA, bna-miR827a, and its target genes were affected in wax-deficient rapeseeds. These results will promote the study of post-transcriptional regulation mechanisms of wax biosynthesis in *B. napus* and provide new directions for further research.

## Introduction

The plant cuticle is a hydrophobic layer covering the surface of aerial organs, which serves a barrier against a variety of environmental changes, such as drought stress, osmotic stress, ultraviolet light, extreme temperature stress, physical damage, dust pollution, and pest and pathogen attacks. In addition, it forms a boundary between organs and plays an important role in pollen fertility and pollen-stigma interactions^1–3^. The plant cuticle consists of two main components, wax and cutin. It is a composite structure with very-long-chain fatty acids (VLCFAs; C20–C34) and their derivatives, including primary alcohols, wax esters, aldehydes, alkanes, primary alcohols, and ketones^4^. In *Arabidopsis thaliana*, the substrates of cutin and wax, C16 or C18 fatty acids from plastids, are activated by long-chain acyl-coenzyme A synthase (LACS) and then exported to endoplasmic reticulum (ER) for further elongation^5^. After adding two carbons by the fatty acid elongase (FAE) complex in each reaction cycle, the ultimate carbon chain length of VLCFAs reaches 20–34^6^. Next, acyl-reduction pathway produces primary alcohols and wax esters, and the decarbonylation pathway produces aldehydes, alkanes, secondary alcohols, and ketones^7^. MAH1, a member of cytochrome P450 enzyme family in *Arabidopsis*, is characterized as a medium-chain alkane hydroxylase, which can catalyze the hydroxylation reaction for generating secondary alcohols and ketones in stem cuticular wax^8^. These cuticular wax components are transported through plastid membrane to plant surface to form wax films or wax crystals, and this process requires ATP to provide energy. *ABCG11* and *ABCG12*, encoding two ATP-binding cassette transporters, are responsible for wax trans-membrane transport in *Arabidopsis*^9^.

Small RNAs are a class of 20–24 nt RNAs, which include two main categories, 20–22 nt microRNAs (miRNAs) and 21–24 nt small interfering RNAs (siRNAs). These small RNAs can regulate gene expression through transcriptional gene silencing (TGS) or post-transcriptional gene silencing (PTGS)^10–12^. RNA polymerase II is responsible for the transcription of miRNA genes to produce hairpin-structured pri-miRNAs. These pri-miRNAs are recognized by RNase III family enzymes to generate pre-cut miRNAs and then cut by DICER-LIKE1 (DCL1) enzyme to produce miRNA duplex complex. Subsequently, the mature miRNAs are loaded into ARGONAUTE 1 (AGO1) to form an active RNA-induced silencing complex (RISC) and target mRNAs with complementarity to the miRNA^13–16^. As for siRNA biosynthesis pathway, several unique proteins are involved in driving specific functions. SUPPRESSOR OF GENE SILENCING3 (SGS3) binds single strand RNAs to prevent them from degradation. RNA-DEPENDENT RNA POLYMERASE1 (RDR1) converts them to double-stranded (ds) RNAs, as a substrate for DCL complex^17,18^. In addition, in *Arabidopsis thaliana*, siRNA-mediated target gene degradation is dependent on SUPERKILLER proteins, which are responsible for RNA degradation in the cytoplasm in the 3ʹ to 5ʹ direction^19^.

In plants, miRNAs participate in diverse aspects of developmental processes and also regulate the response to environmental stresses. For example, in *Arabidopsis*, miR156 and miR172, cooperate to regulate the transition from the juvenile to the adult phase^20^. Another conserved miRNA, miR319, controls leaf morphology and size by targeting TEOSINTE BRANCHED1/CYCLOIDEA/PCF (TCPs) in tomato^21^. Recently, several studies have found that small RNAs participate in cuticular wax biosynthesis. In *Arabidopsis*, *CER7* encodes a 3ʹ–5ʹ exoribonuclease and degrades a *CER3*-recognized ta-siRNA to regulate *CER3* expression^22,23^. This *CER3* regulation process is dependent on RNA-mediated gene silencing components (RDR1 and SGS3), and the SUPERKILLER complex components, AtSKI2, AtSKI3 and AtSKI8^24,25^. In durum wheat, *W1-COE* encodes a carboxylesterase to participate in the *β*-diketone biosynthesis process. The *IW1* gene produces a miRNA to cleave *W1-COE* and *W2-COE* transcripts, which results in *β*-diketone decrease and non-glaucous phenotype^26^.

*Brassica napus* is a major vegetable oil-producing crop worldwide. Small RNA sequencing technology has helped to identify small RNAs in different biological processes and phenomena in rapeseed, including oil-production^27^, seed maturation^28^, pollen development^29^ and hybrid vigor^30^. In addition, miRNAs play significant roles in stress response, such as cadmium stress^31^, *Plasmodiophora brassicae* attacks^32^, and *Verticillium longisporum* attacks^33^. Recently, *B. napus* genome has been published^34^. The rapeseed genome sequence information, together with small RNA sequencing technology, provides a convenient strategy for studying miRNA expression and function in *B. napus*.

Previously, most studies have investigated the gene-participated and transfactor-regulated wax biosynthesis pathway in many plants. However, very few studies on miRNA-involved regulation of wax synthesis in *B. napus* have been reported to date. In our previous study, a dominant glossy mutant of rapeseed was studied^35^. We assumed that there might be a dominant repressor to inhibit wax-related gene expression. To verify this hypothesis and explore the function of miRNAs in wax biosynthesis, we used small RNA sequencing technology in our current study. In *Arabidopsis*, the elongation part with the fastest rate is on the top 3 cm of the stem. The biosynthesis and deposition of cuticular wax are constant during cell elongation, which means that the wax-related genes are highly expressed in the epidermal cells of this segment^36^. We peeled the epidermis of *B.napus* manually from the top 3 cm of the stem at four weeks after cultivation. Subsequently, four small RNA libraries (i.e., two replicates for each phenotype) were constructed for glossy mutant and wild-type stem epidermis and then sequenced using Illumina Hiseq 2000 platform. The aim of this work was to identify the differentially-expressed miRNAs between glossy and waxy rapeseed, and to find out their potential function in wax biosynthesis. These results will help to elucidate the mechanism by which small RNAs are involved in wax biosynthesis regulation in *B. napus*.

## Results

### Phenotypic characterization of glossy and waxy plants

**Figure 1:**
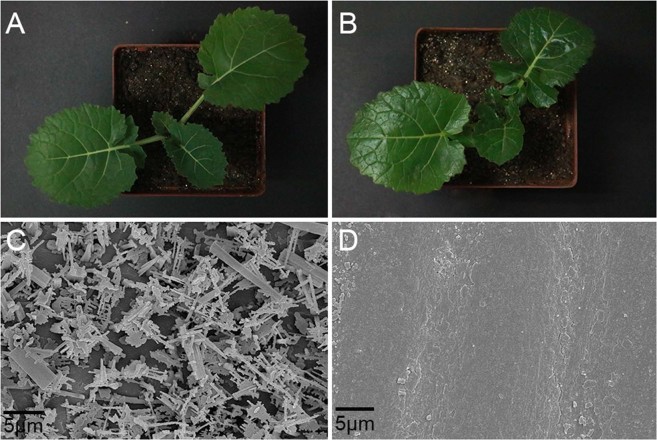
Phenotypic characterization of glossy and waxy epidermis. (**A**) Phenotype of wild-type plant. (**B**) Phenotype of glossy plant. (**C**) Wax crystals on wild-type stem are abundant and intact. **(D)** Epidermis of glossy stem is smooth, with nearly no wax crystals. Scale bar = 5 μm.

The wax-deficient rapeseed material is a novel dominant glossy mutant, as described previously^35^. The wild-type material (NO.2127) was glaucous with all the organs above ground covered with wax (Fig. 1A), while the glossy mutant (P4) was observed to have bright green leaves and stems (Fig. 1B). Cryogenic scanning electron microscopy (Cryo-SEM) was used to examine the deposition of epicuticular wax crystals on the stem surface. The wild-type stem was covered with abundant wax crystals (Fig. 1C). In contrast, nearly no visible wax crystals could be seen on the stem cuticle (Fig. 1D).

### Basic analysis and length distribution of small RNA

**Figure 2:**
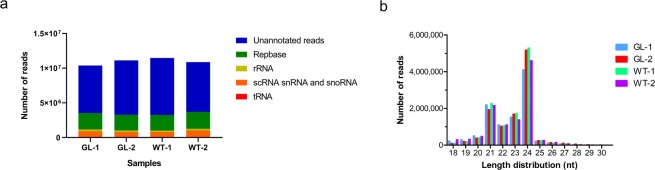
Annotation distribution and length distribution of diverse clean reads. **(a)** All the clean reads were classified into five groups. The blue columns represent unannotated reads and account for more than 60% in each library. The y-axis represents reads number of each term. **(b)** The length distribution of clean reads in each library. The 24nt sequences represent the dominant percentage, following by 21nt. The x-axis represents different length and the y-axis represents the reads number.

In order to explore the potential miRNAs participating in the regulation of wax biosynthesis in *B. napus*, four independent sRNA libraries (two biological replicates for each phenotype) were constructed for sequencing by Illumina HiSeq 2000. After filtering low quality tags, < 18 nt or > 30 nt reads, 3′ adapter, insert sequence, 5′ adapter and polyA, 43,840,451 clean reads were generated (Table 1). These clean reads contained tRNA, scRNA, snRNA, snoRNA, rRNA, repeat sequences and un-annotated reads. The reads distribution in these four small RNA libraries showed extreme similarity with each other (Fig. 2a). Among the clean reads, 27,463,633 were un-annotated, accounting for approximately 62.5% of all clean reads (Table 1). Subsequently, all the above un-annotated reads were mapped to the *B. napus* genome with a tolerance of two mismatches. More than 44% (>3 million reads in each library) of the un-annotated reads could be mapped to the reference genome (Supplementary Table S1). These un-annotated reads were submitted to miRBase 21.0 to search known miRNAs in all plants, and only 2,628,951 (approximately 6%) reads were identified as conserved miRNAs (Table 1). A majority of small RNA reads ranged from 21–24 nt, with 24 nt sequences representing the dominant percentage (Fig. 2b). These results were consistent with the length distribution patterns of small RNAs in previous studies on plants^29,37^.

**Table 1:** Summary of small RNA sequencing data.

Samples	GL-1	GL-2	WT-1	WT-2	Total
Raw reads	11,409,851	12,072,823	12,444,196	12,144,309	48,071,179
Low quality reads	907,530	928,213	933,421	988,058	3,757,222
<18nt and >30nt reads	125,250	23,690	36,441	288,125	473,506
Clean reads	10,377,071	11,120,920	11,474,334	10,868,126	43,840,451
Conserved miRNA	663,944	544,234	720,981	699,792	2,628,951
Conserved miRNA percentage	6.40%	4.89%	6.28%	6.44%	—
Unannotated reads	6,183,278	7,300,046	7,505,286	6,475,023	27,463,633
Unannotated percentage	59.59%	65.64%	65.41%	59.58%	—

### Identification of conserved and novel miRNAs

**Figure 3:**
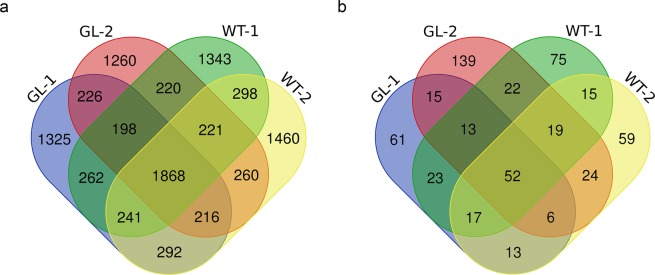
Overlap of reads mapped to known miRNAs and novel miRNAs in four libraries. **(a)** Statistics of known miRNA reads in four small RNA libraries. **(b)** Comparison of novel miRNAs in four small RNA libraries.

To date, the miRBase 21.0 contained a total of 35,828 mature miRNAs and 28,645 miRNA precursors from different types of plants (http://www.mirbase.org/). Totally, we identified 9,690 unique reads which could mapped to conserved miRNAs in miRBase 21.0. Only 1,868 reads were co-expressed in four small RNA libraries, and the number of unique reads in glossy and wildtype materials were 2,811 and 3,101, respectively (Fig. 3a). Subsequently, in the four libraries, 300 unique miRNAs from 170 families were examined, including 199 *Brassica* miRNAs from 77 families (Table 2). Among these conserved miRNA families, the three most-abundant miRNA families (reads ≥ 120,000) were of miR157, miR156, and miR158 (Supplementary Table S2). We also predicted novel miRNAs using MIREAP software to identify more miRNAs, which might exist in the four libraries. All the reads that were not annotated as known small RNAs, but mapped to the *B. napus* genome, were used to detect novel miRNAs. In these four libraries, 553 unique novel miRNAs (reads ≥ 10) were identified (Table 2). The number of common novel miRNAs in these four libraries was 52, which only constituted a small part of all the novel miRNAs. The number of unique novel miRNAs in these four libraries were 61 (GL-1), 139 (GL-2), 75 (WT-1), and 59 (WT-2), respectively (Fig. 3b).

**Table 2:** The number of conserved and novel miRNAs in four libraries.

	Numbers
Known miRNAs	300
Known miRNA families	170
Brassica miRNAs	199
Brassica miRNA families	77
Novel-miRNAs	533

### Analysis of differentially expressed miRNAs

**Figure 4:**
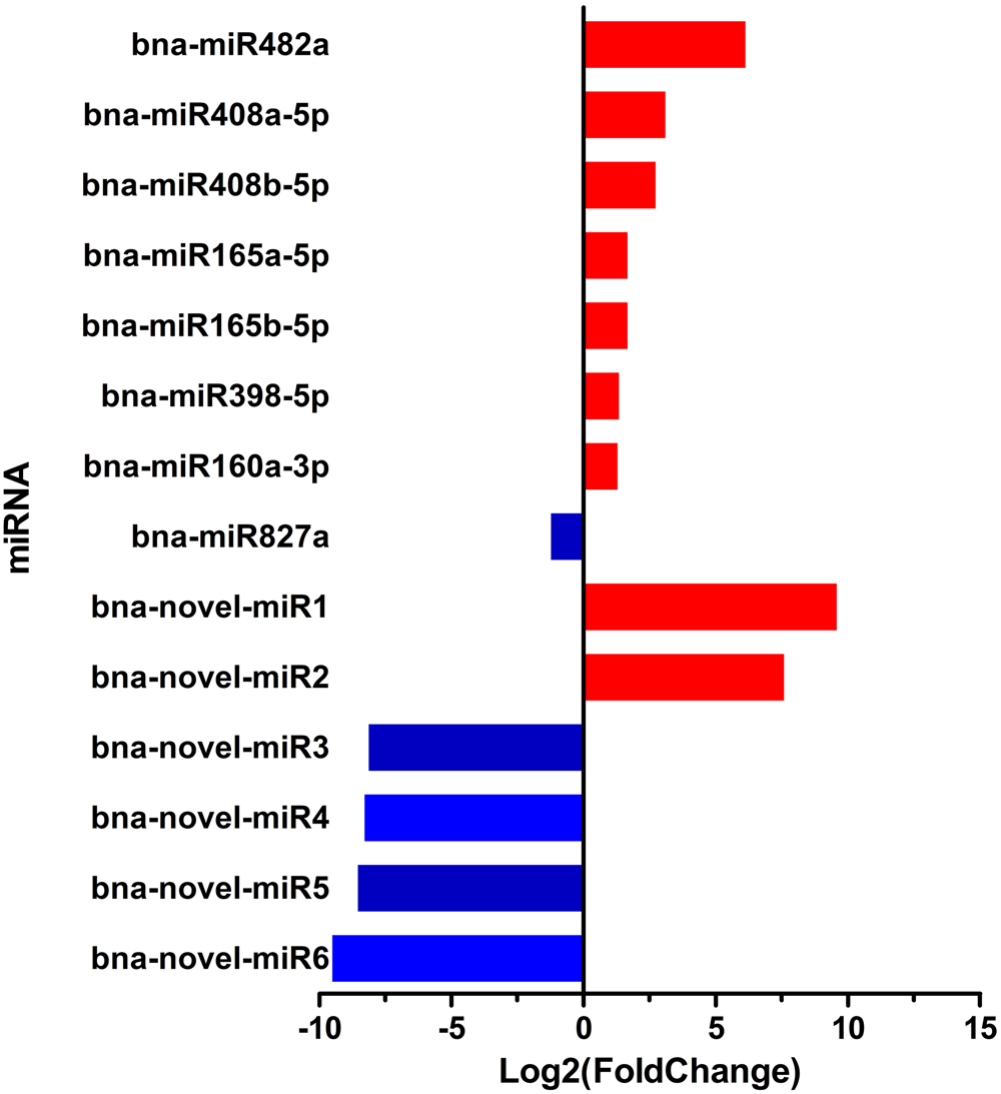
Differentially-expressed miRNAs in four libraries. The red columns represent up-regulation and the blue columns represent down-regulation. The x-axis represents log_2_(Fold change).

Among these 300 conserved miRNAs, eight were differentially expressed (|log_2_(Foldchange)| ≥ 1 and Statistical *P* < 0.05), including seven up-regulated miRNAs (bna-miR482a, bna-miR408a-5p, bna-miR408b-5p, bna-miR165a-5p, bna-miR165b-5p, bna-miR398-5p, and bna-miR160a-3p) and one down-regulated miRNA (bna-miR827a). These eight miRNAs belonged to six miRNA families. In addition, six novel miRNAs showed differentially expressed, with two up-regulated novel-miRNAs (bna-novel-miR1 and bna-novel-miR2) and four down-regulated novel-miRNAs (bna-novel-miR3, bna-novel-miR4, bna-novel-miR5, and bna-novel-miR6) (Fig. 4).

Subsequently, we analyzed the sequence of these differentially expressed miRNAs. The sequence of bna-miR408a-5p was similar to that of bna-miR408b-5p, with one adenine lacking at the 5ʹ end and one additional guanine at the 3ʹ end. Compared to that of bna-miR165a-5p, the sequence of bna-miR165b-5p contained only one additional guanine at the 5ʹ end (Table 3). After comparing the sequence of these differentially expressed miRNAs with that of the reported miRNAs, we found that bna-miR408b-5p, bna-miR165b-5p, bna-miR160a-3p, and bna-miR398-5p were all complementary to their mature strands (Table 3, Supplementary Table S3).

**Table 3:** The sequence of differentially expressed miRNAs.

miRNA	Sequence
bna-miR482a	AGAUGGGUGGCUGGGCAAGAAG
bna-miR408a-5p	CAGGGAACAAGCAGAGCAUGG
bna-miR408b-5p	ACAGGGAACAAGCAGAGCAUG
bna-miR165a-5p	GAAUGUUGUCUGGAUCGAGG
bna-miR165b-5p	GGAAUGUUGUCUGGAUCGAGG
bna-miR398-5p	GGGUCGACAUGAGAACACAUG
bna-miR160a-3p	GCGUAUGAGGAGCCAUGCAUA
bna-miR827a	UUAGAUGACCAUCAACAAACA
bna-novel-miR1	AGACUGGAGUAGUGAAAUGAUGG
bna-novel-miR2	AGAGUUGAUCGACUGACUCCA
bna-novel-miR3	AGGCAGUUGUAUCUUGGGAUUCUU
bna-novel-miR4	GUGGGGUGAUCCGGCGACCGUGGU
bna-novel-miR5	ACUACUGUAGUAGCGUCGAUCGAC
bna-novel-miR6	GCAGUCGGCGAAACUUGCGUGGGC

### Validation of differentially expressed miRNAs

**Figure 5:**
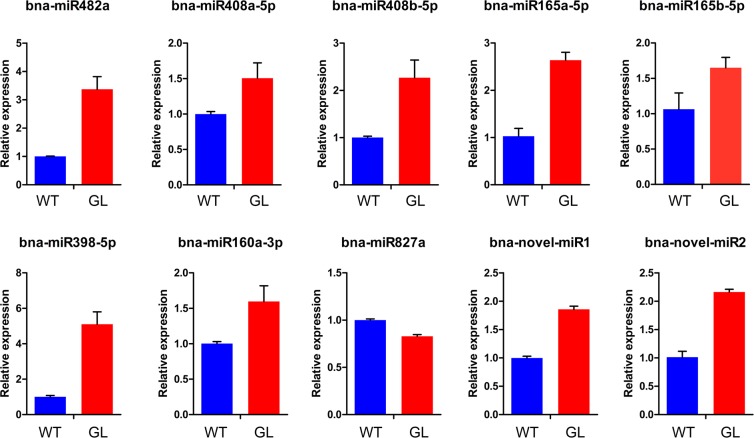
Validation of differentially-expressed miRNAs by stem-loop qRT-PCR. The bars indicate standard error (SE) of the mean (*n* = 3).

To confirm the results of small RNAs sequencing experiment, total RNA extracted from glossy and waxy stems was subjected to stem-loop qRT-PCR. In total, we examined the expression of seven up-regulated conserved miRNAs (bna-miR482a, bna-miR408a-5p, bna-miR408b-5p, bna-miR165a-5p, bna-miR165b-5p, bna-miR398-5p, and bna-miR160-3p), one down-regulated miRNA (bna-miR827a), and two novel-miRNAs (bna-novel-miR1 and bna-novel-miR2). All the above miRNAs had consistent expression trends with the sequencing data, even though the differential expression levels were not equal. In the stem-loop qRT-PCR experiments, the expression level of bna-miR482a in the glossy mutant was approximately four times higher, compared to the wild type, whereas the expression level estimated by sequencing changed by approximately twelve times (Fig. 5).

### Target prediction of different expressed miRNAs

In order to analyze the function of differentially expressed miRNAs, we used the psRNATarget (http://plantgrn.noble.org/psRNATarget/analysis) software to identify putative target genes. As a result, 10 genes were identified as the potential targets of four differentially-expressed conserved miRNAs. These potential targets included peptide chain release factor 1, leucine-rich repeat protein kinase, *CYP96A2*, tonoplast monosaccharide transporter 3 and SPX domain-containing protein (*NAL*). Except for bna-miR827a targets, the rest of the predicted genes were all novel putative targets of these conserved miRNAs in *B. napus*. As for the novel miRNAs, only bna-novel-miR2 and bna-novel-miR3 had predicted target genes. *BnaC07g31010D*, encoding a zinc ion-binding protein, was the predicted target of bna-novel-miR2. Bna-novel-miR3 was predicted to target three genes with two of them being proteins of unknown function (Table 4).

**Table 4:** Functional annotation of predicted target genes of differentially-expressed miRNAs.

miRNA	Target gene	*A. thaliana* homologous gene	Target site^a^		Target gene description
			Start	End	
Bna-miR408a-5p	BnaC04g00470D	AT2G47020	1519	1539	Peptide chain release factor 1
	BnaC09g28020D	AT5G51560	2248	2268	Leucine-rich repeat protein kinase family
Bna-miR165a-5p	BnaC01g28580D	—	134	153	Protein of unknown function
	BnaA06g40560D	AT4G32170	293	313	CYP96A2
Bna-miR160a-3p	BnaA09g32040D	AT3G51490	1492	1512	Tonoplast monosaccharide transporter 3
	BnaC08g22860D	AT3G51490	1528	1548	Tonoplast monosaccharide transporter 3
Bna-miR827a	BnaC08g45940D	AT1G02860	95	115	SPX (SYG1/Pho81/XPR1) domain-containing protein
	BnaA09g51130D	AT1G02860	79	99	SPX (SYG1/Pho81/XPR1) domain-containing protein
	BnaA10g01450D	AT1G02860	32	52	SPX (SYG1/Pho81/XPR1) domain-containing protein
	BnaC05g01480D	AT1G02860	160	180	SPX (SYG1/Pho81/XPR1) domain-containing protein
Bna-novel-miR2	BnaC07g31010D	AT5G61120	91	113	Zinc ion-binding protein
Bna-novel-miR3	BnaC08g08850D	AT4G13780	15	38	Methionine–tRNA ligase
	BnaC04g33280D	AT2G21720	2720	2743	Protein of unknown function
	BnaC04g08980D	—	462	485	Protein of unknown function

^a^Nucleotides to the 5′ end.

### Experimental validation of predicted targets and their expression profiling

**Figure 6:**
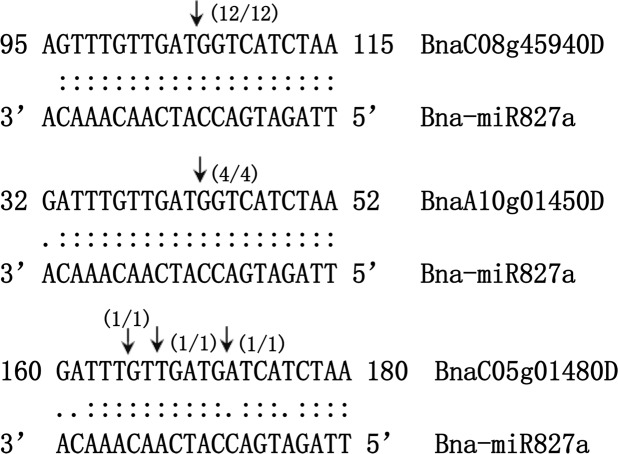
The verified cutting sites of bna-miR827a on its target genes by 5′ RACE in *Brassica napus*. *BnaC08g45950D*, *BnaA10g01450D* and *BnaC05g01480D* genes are the predicted targets of bna-miR827a. The cleavage sites of *BnaC08g45950D* and *BnaA10g01450D* are precisely mapped from the 10^th^ position of the complement of bna-miR827a 5′ end. Three different breakpoints on *BnaC05g01480D* were detected by 5′ RACE, which are broken at the 9^th^, 14^th^, and 16^th^ position from bna-miR827a 5′ end, respectively.

To identify the cleavage products of the predicted target genes in *B.napus*, 5′ RACE experiments were performed. As a result, the cleavage sites of *BnaC08g45950D* and *BnaA10g01450D* were precisely mapped from the 10^th^ position of the complement of bna-miR827a 5′ end. Surprisingly, although *BnaC05g01480D* could be cleaved by bna-miR827a, the cutting site was not conserved. We have detected three different cleavage products of *BnaC05g01480D*, and the breakpoints were at the 9^th^, 14^th^, and 16^th^ positions of bna-miR827a, respectively (Fig. 6). Unfortunately, no other target genes could be detected to be cleaved by their corresponding miRNAs.

**Figure 7:**
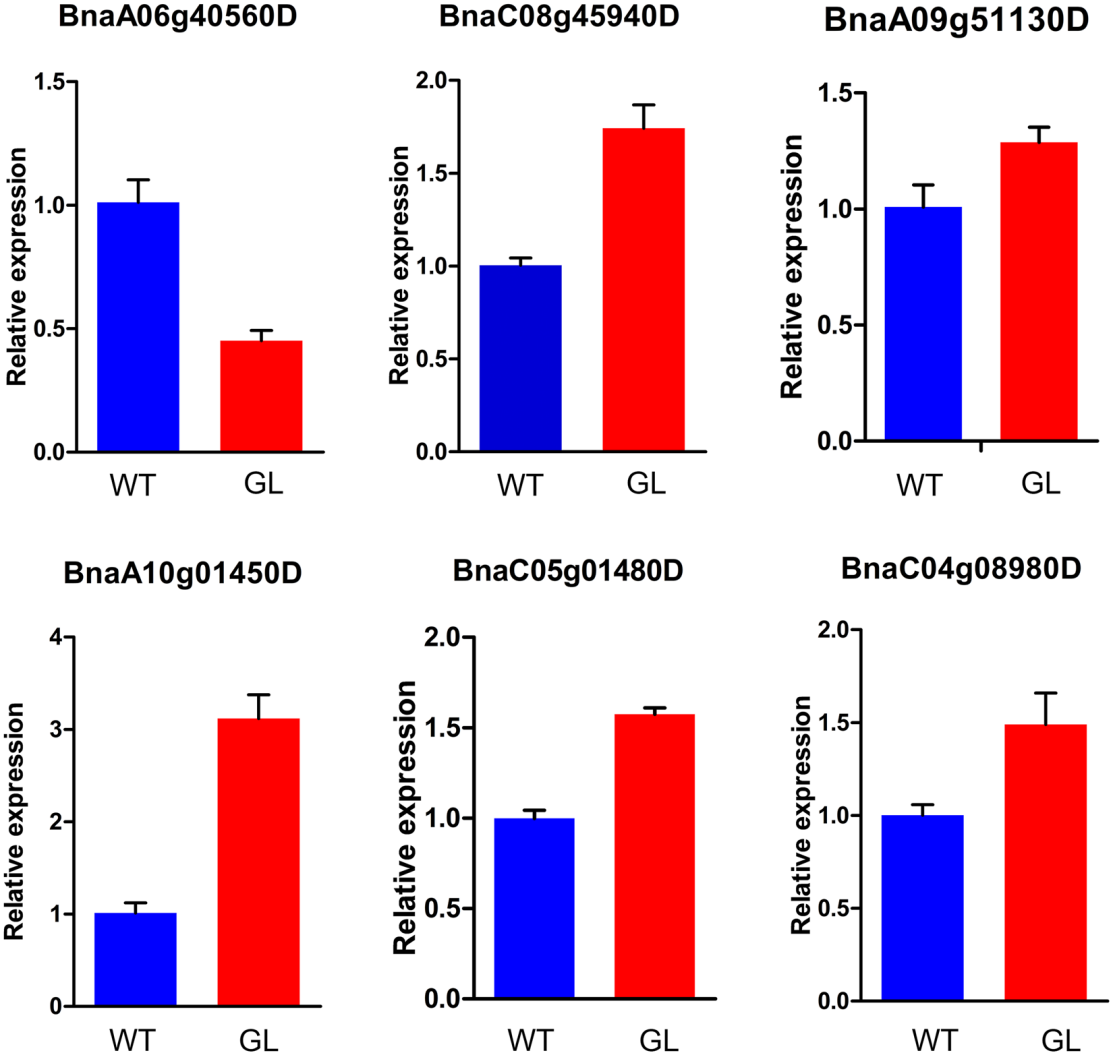
Relative expression level change of predicted target genes verified by qRT-PCR in wild-type and mutant plants. The bars indicate SE of the mean.

In order to further assess the effects of miRNAs on predicted target genes in mutant and wild-type samples, qRT-PCR was employed to analyze the expression of these genes with the expectation that targets would be negatively regulated by corresponding miRNAs. The expression levels of fourteen predicted target genes were compared between glossy stem and wild-type stem. One up-regulated miRNA, Bna-miR165a-5p, was predicted to target two genes in rapeseed, *BnaC01g28580D* and *BnaA06g40560D*. Through qRT-PCR detection, we found that *BnaA06g40560D* was down-regulated in glossy mutant. Meanwhile, the decreased expression of bna-miR827a in the mutant samples might have reduced cleavage of its targets, as the expression pattern of *BnaC08g45940D*,*BnaA09g51130D*, *BnaA10g01450D* and *BnaC05g01480D* genes negatively correlated with the expression of bna-miR827a. Similarly, the down-regulation of bna-novel-miR3 correlated with the up-regulation of the predicted target *BnaC04g08980D* in the glossy mutant (Fig. 7). The relative expression levels of the rest eight genes did not show opposite tendency with their corresponding miRNAs (Supplementary Fig. 1).

## Discussion

The wax-deficient mutant used in this research showed glossy phenotype during the whole growth period and was easily detectable with naked eyes. In *Arabidopsis*, the epidermis of stems is covered by different types of wax crystals, such as rods, filaments, tubes and plates^6^. Cryo-SEM showed that there were almost no classic wax crystals the stems of mutant (Fig. 1). Transmission electron microscopy (TEM) and Toluidine Blue stain experiments have proved that the cuticle structure of mutant leaf and stem was destroyed^35^. In recent years, several cuticle-related genes have been cloned in genus *Brassica* by forward genetic approaches, including *Brcer1* in non-heading Chinese cabbage^38^, *Cgl1* (*CER1*) and *Cgl2* (*CER4*) in *Brassica oleracea*^39,40^. However, all of these glossy phenotypes were recessive mutants, different from our dominant mutant.

miRNA are post-transcriptional regulators of gene expression through repressing translation or directing degradation of their target mRNAs, which have been found in all plants and animals^41^. *B.napus* is one of the major edible oil crops worldwide, and the genome sequence is now available^34^. To identify wax-related miRNAs in *B.napus*, four small RNA libraries were constructed and sequenced. A total of 43,840,451 clean reads were obtained and those unannotated reads were mapped to conserved miRNAs of all plants in miRBase 21.0 database. Subsequently, we identified 300 conserved miRNAs in total and eight of them showed differential expression (Table 2, Fig. 2). To our surprise, bna-miR408b-5p, bna-miR165b-5p, bna-miR160-3p and bna-miR398-5p were all complementary strands of corresponding mature miRNAs published previously (Table 3, Supplementary Table S3).

The function of mature strands of these different-expressed miRNAs have been reported in *Arabidopsis*. miR408 is reported to target six genes (*CUPREDOXIN*, *UCC2*, *LAC3*, *PLANTACYANIN*, *LAC12* and *LAC13*) in *Arabidopsis* and to play important roles in response to senescence, low copper and other abiotic stresses^42–44^. miR165 is a relatively abundant miRNA in *Arabidopsis*. It regulates several HD-ZIP III transcriptional factors to determine the cell fate of adaxial–abaxial patterning in leaf tissues and xylem differentiation in root stele tissues^45^. In *Arabidopsis*, miR160 targets three *AUXIN RESPONSE FACTORS* (*ARF16*, *ARF17* and *ARF10*) to regulate the development of root, leaf, and flower organs, seed germination, and post-germination changes^46^. In addition, miR160 is also a significant signaling element that maintains the stage-specific auxin/cytokinin balance to direct proper nodule formation and maturation in soybean^47^. In terms of stress response, miR398 plays important roles in response to senescence, oxidative stress, low copper and heat stress^48,49^. According to our results, we hypothesized that these differentially-expressed complementary strands detected in our research in glossy mutant might have different functions from their corresponding conserved strands.

In general, during miRNA biogenesis, only one miRNA strand (also named as mature miRNA) is loaded onto an AGO protein to form RISC complex, while a complementary strand (also named as passenger strand) is subsequently degraded^50,51^. However, in *Arabidopsis*, there are ten AGO proteins in total. Each AGO complex recruits one or two kinds of small RNAs with specific 5ʹ-terminal nucleotide. miR319 and miR393b with U at 5ʹ end are incorporated into the AGO1 complex while their complementary strands with 5ʹ-terminal A are loaded onto AGO2^52^. In *Drosophila*, AGO1 preferentially loads miRNA strand to regulate target gene expression, whereas AGO2 often recruits the miRNA complementary strand. This indicates that each miRNA precursor generates two mature small RNA strands. These two strands participate in different RNAi pathways, which further implicates that miRNA complementary strands also have significant regulatory function^12,53^. In *B. napus*, the complementary strands of miR160, miR171b, and miR408 showed extremely high expression levels compared to their mature miRNA strands, and the complementary strand of miR408 might have some function in fatty acid biosynthesis^27^. All the above studies indicated that miRNA complementary strands also participate in the post-transcriptional regulation of gene expression.

One of the predicted target genes of bna-miR165a-5p was *BnaA06g40560D*. The CDS of *BnaA06g40560D* (1557 bp) contains only one exon. It encodes a protein containing 518 amino acids (Supplementary Fig. 2A). Subsequently, we searched the possible conserved domains existed in the protein sequence of *BnaA06g40560D* in NCBI website (https://www.ncbi.nlm.nih.gov/cdd). The protein sequence of *BnaA06g40560D* contains the conserved domain of P450 superfamily (Supplementary Fig. 2B). The CDS of *BnaA06g40560D* was then submitted to NCBI to search the homologous genes in other plants (https://blast.ncbi.nlm.nih.gov/Blast.cgi). *AT1G57750* is the homologous gene of *BnaA06g40560D* in *Arabidopsis*. The protein sequence of *BnaA06g40560D* is 76% identical to the *AT1G57750* amino acids. *AT1G57750* is a member of the CYP96A family. It encodes a mid-chain-hydroxylase1 enzyme (MAH1) and is involved in the biosynthesis of secondary alcohols and ketones. The mutant of *mah1* resulted in lower levels of secondary alcohols and ketones, while with increased alkane amount in *Arabidopsis*^8^. In our previous study, wax compositional analysis found that ketones and secondary alcohol content severely decreased in our glossy rapeseed mutant, indicating that ketones and secondary alcohol biosynthesis-related genes might be down-regulated^35^. As *BnaA06g40560D is* the homolog of *AT1G57750*, we speculated that *BnaA06g40560D* might participate in the biosynthesis of ketones and secondary alcohol in *B. napus*. In our research, qRT-PCR data also confirmed that the expression level of *BnaA06g40560D* decreased in glossy mutant. We, therefore, hypothesized that the up-regulation of bna-miR165a-5p repressed the expression of *BnaA06g40560D*, thereby leading to the decrease of secondary alcohol and ketone content. The function of bna-miR165a-5p and *BnaA06g40560D* need to be further investigated in rapeseed. miR827 was another interesting miRNA, which was down-regulated in our wax-deficient material. The development of plants is influenced by many environmental factors, such as nutrient availability, drought stress and biotic attack. miR827 was reported to drive different functions in response to environmental stresses. In *Arabidopsis* and rice (*Oryza sativa*), miR827 is upregulated under Pi deficiency stress^54,55^. In most angiosperms, miR827 conservatively targets *PHT5* homologs, but in *Brassicaceae* and *Cleomaceae* it preferentially targets *NLA* homologs^56^. *NLA* is the target of miR827 in *Arabidopsis*. It encodes a plasma-membrane-associated RING-type ubiquitin E3 ligase with an N-terminal SPX domain and controls Pi acquisition by ubiquitinating PHOSPHATE TRANSPORTER 1 (PHT1) family members^54,57^. However, in rice, miR827 targets two members of the PHOSPHATE TRANSPORTER 5 (*PHT5*) family, which function as Pi storage or remobilization^55,58^. Besides the function related to Pi utilization, miR827 also mediates plant susceptibility to *Heterodera schachtii* in *Arabidopsis*^59^. In addition, in barley, over-expression of Hv-miR827 could enhance drought tolerance^60^. These results indicate that miR827 is a factor in response to many environmental changes. In our previous study, we found that the glossy mutant showed higher water-loss rates when compared with wild-type materials^35^. Wax-deficiency on leaves and stems results in increased sensitivity to drought stress in glossy mutant. We suppose that the expression of many genes, especially those related to environmental stresses, may be influenced in glossy mutant. miR827 might be one of important miRNAs which showed down-regulation due to wax deficiency in rapeseed. In our research, 5′ RACE experiments showed that miR827 could cleave three NLA genes (*BnaC08g45940D*, *BnaA10g01450D*, and *BnaC05g01480D*). The relative expression levels of these three target genes were up-regulated in the glossy mutant samples. We hypothesized that down-regulation of miR827 was due to the wax-deficiency of the mutant samples, but the relationship and mechanism regarding wax biosynthesis and miR827 expression need to be further studied.

In conclusion, for the first time, we found that bna-miR165a-5p seems to participate in the wax biosynthesis process by regulating its putative target *BnaA06g40560D*. The expression level of a Pi-related miRNA, bna-miR827a, and its target genes were affected in wax-deficient rapeseeds. In future studies, it will be interesting to investigate the function of bna-miR165a-5p and bna-miR827a in *Brassica napus*.

## Materials and Methods

### Plant materials and sample collection

The wax-deficient rapeseed material (*Brassica napus* L. P4) was backcrossed with a wild-type material (NO. 2127) to develop a BC_5_ population, which was cultivated in the experimental field in Huazhong Agricultural University (Wuhan, Hubei Province, China). P4 is a natural mutation material discovered in the field of Chengdu Academy of Agriculture and Forestry Science, whereas NO. 2127 is a resynthesized *B. napus* line with intact cuticle structure. The wild-type material (NO. 2127) was used to cross with glossy material (P4) to generate F_1_ individuals. All of the F_1_ individuals showed glossy phenotype, indicating that the glossy trait was dominant. The stem epidermis from six independent individuals of glossy progeny and six independent individuals of waxy progeny were peeled at four weeks after cultivation. These harvested materials were stored in liquid nitrogen for subsequent RNA isolation.

### RNA extraction and library construction

Total RNA was extracted using TRIzol reagent (Invitrogen, Carlsbad, CA, USA) according to the manufacturer’s instructions. RNA quality and quantity were measured with the NanoDrop 2000 (Thermo Scientific, Waltham, MA, USA) and Agilent 2100 Bioanalyzer (Agient, Santa Clara, CA, USA). After RNA being extracted, equal quantities of three glossy or wild-type RNA samples were pooled and named GL-1, GL-2, WT-1, and WT-2, respectively. For each phenotype, two biological replicates were sequenced for further analysis.

The small RNA library construction was carried out according to the following steps. Firstly, 18–30 nt small RNAs were isolated from four total RNA libraries by size fractionation with 15% polyacrylamide gel, and ligated with 5ʹ- and 3ʹ-RNA adapters. Secondly, reverse-transcription PCR using Super-Script II Reverse Transcriptase was used to create cDNA constructs based on two adapters, followed by PCR amplification. Finally, the PCR products were purified and sequenced by Illumina Hiseq 2000 (Beijing Genomics Institute, Shenzhen, China).

### Small RNA sequencing data analysis

After small RNA sequencing, the raw reads were filtered to remove low-quality reads, adaptor sequences, <18 nt and >30 nt reads, and other containments using the Fastx-toolkit (http://hannonlab.cshl.edu/fastx_toolkit/). Thereafter, the clean reads were blasted using Rfam database (10.1) and the GenBank noncoding RNA database to remove ribosomal RNAs (rRNAs), transfer RNAs (tRNAs), small nuclear RNAs (snRNAs), small nucleolar RNAs (snoRNAs), repeat sequences, and other non-coding RNAs (ncRNAs). The remaining high-quality reads were aligned to *B. napus* genome (http://www.genoscope.cns.fr/brassicanapus/) with SOAP software (http://soap.genomics.org.cn/), and then those perfect-matched reads were submitted to the miRBase 21.0 (http://www.mirbase.org) database to identify known miRNAs with a criteria for two nucleotide mismatches in all plant miRNAs.

The remaining reads not identified as conserved miRNAs were used to predict novel miRNAs. We mapped these novel reads to reference genome to find their flanking sequences and folded the regions to a hairpin structure between the mature miRNA-5p and miRNA-3p sequence on the opposite hairpin arm. The novel miRNAs satisfied the following criteria: the hairpin structure had free energy lower than −18 kcal mol^−1^; the space between 5p and 3p miRNA sequences was less than 300 nt; the space between 5p and 3p miRNA sequences was more than 16 matched nucleotides and the number of nucleotide bulges between 5p and 3p miRNAs fewer than four; the miRNA-5p/miRNA-3p duplex was less than one, with a 2 nt 3′ overhang.

### Analysis of differentially-expressed miRNAs and target genes

The expression level of each miRNA was normalized using fragments per kilobase million (FPKM) methods. Thereafter, DESeq package in R was used to identify differentially expressed miRNAs between the glossy and wild-type libraries. The differentially-expressed miRNAs should satisfy the following criteria: |log_2_(foldchange)| ≥ 1 and Statistical *P* < 0.05. Mostly, the target sites on target genes have sequence complement character with corresponding miRNA sequence^12^. Those up-and down-regulated miRNA sequences were submitted to the target prediction website (http://plantgrn.noble.org/psRNATarget/analysis), in order to identify potential target genes in *B. napus*. Subsequently, these predicted target genes in rapeseed were annotated according to corresponding homologous genes in *Arabidopsis*, using the TAIR website (http://www.arabidopsis.org/index.jsp).

### 5′-rapid amplification of cDNA ends (5′ RACE)

The SMARTer RACE 5′/3′ Kit (Clontech) was used to verify the precise cleavage sites of predicted target genes, following the manufacturer’s instructions. Briefly, two antisense gene-specific primers (GSPs) were designed according to the gene sequences. Then, samples with 5 μg of high quality total RNAs were prepared for further reverse transcription reactions. Subsequently, two nested GSPs and a universal primer mix (UPM) were used for each amplification of 5′ end cDNAs. The positive 5′ RACE amplification products were gel-purified and cloned into the PMD18-T vector for sequencing. For each gene, at least 10 independent clones were sequenced. All the primers used in this experiment are listed in Supplementary Table S4.

### Verification of miRNA and target gene expression by stem-loop qRT-PCR and qRT-PCR

The stem-loop qRT-PCR method was used to verify the expression levels of miRNAs according to previously reported procedures^50^. In the stem-loop qRT-PCR experiments, 2 μl total RNA was used to synthesize cDNA using the First Strand cDNA Synthesis Kit (Thermo Scientific, Lithuania, EU) with stem-loop reverse transcript (RT) primers and oligo (dT)_18_ primers. Differentially-expressed miRNAs were validated using SYBR Green II (Toyobo, Osaka, Japan) by a Bio-Rad CFX96 according to the manufacturer’s protocol. PCR was performed using 8.4 μl of 20 × diluted cDNA products, 10 μl 2× SYBR Green II buffer, and 0.8 μl forward and reverse primers (10 μM) in each 20 μl system. For each miRNA, a specific forward primer (FP) and a universal primer (UP) was used in PCR. *BnActin* gene was used as internal control for each reaction. The reactions were performed at 95 °C for 10 s, followed by 45 cycles of 5 s at 95 °C, 10 s at 60 °C, and 30 s at 72 °C, and 10 s at 95 °C. All reactions were performed in three independent experiments. The relative expression levels were calculated using the 2 delta-delta Ct method^61,62^. The same cDNA samples were used for examination of relative expression level of predicted target genes. For each phenotype, we manually collected stems from three individuals to test the expression of small RNAs and target genes. Primers used in this step were listed in Supplementary Table S5.

## Supplementary information

Supplementary tables

Supplementary figures

## Data Availability

The datasets generated during the current study are included in this published article and its supplementary information files. The sequencing raw data from this study are available in Gene Expression Omnibus (GEO) repository with the accession number GSE115073 (https://www.ncbi.nlm.nih.gov/geo/query/acc.cgi?acc=GSE115073).
