# Supplementary material for: Differential expression of miRNAs and their targets in wax-deficient rapeseed
Source: Sci Rep. 2019 Aug 21;9:12201. doi: 10.1038/s41598-019-48439-z (PMC6704058; doi:10.1038/s41598-019-48439-z)
Supplement: Supplementary file 1 — Supplementary tables [file 41598_2019_48439_MOESM1_ESM.pdf]

Supplementary tables

Title

Differential expression of miRNAs and their targets in wax-deficient rapeseed

Tingting Liu[1], Jingquan Tang[1], Li Chen[1], Jiayue Zeng[1], Jing Wen[1], Bin Yi[1], Chaozhi Ma[1], Jinxing Tu[1], Tingdong Fu[1], Jinxiong Shen[1]*

[1] National Key Laboratory of Crop Genetic Improvement, National Center of Rapeseed Improvement, Huazhong Agricultural University, Wuhan, Hubei, China

*Corresponding author: Dr. Jinxiong Shen; jxshen@mail.hzau.edu.cn

SUPPLEMENTARY TABLE S1

The number and percentage of unannotated reads mapped to *Brassica napus* genome

Samples	Unannotated reads	Mapped reads	Mapped Percentage	Unmapped reads	Unmapped Percentage
GL-1	6847222	3011671	44.00%	3835551	56.00%
GL-2	7844280	3471579	44.30%	4372701	55.70%
WT-1	8226267	3676372	44.70%	4549895	55.30%
WT-2	7174815	3197561	44.60%	3977254	55.40%

SUPPLEMENTARY TABLE S2

The reads number of top three conserved miRNAs detected in four libraries

	GL-1	GL-2	WT-1	WT-2
miRNA ID	reads number	reads number	reads number	reads number
aly-miR157c-5p	168249	138997	236193	182972
ptc-miR156h	168229	138993	236165	142245
gma-miR156e	168225	138993	236162	142241
cca-miR156c	168224	138991	236162	142240
nta-miR156j	168200	138950	236116	142239
sly-miR156a	168185	138945	236088	142198
nta-miR156i	168180	138923	236082	142174
mtr-miR156f	166723	138010	234311	142174
atr-miR156a	166722	138008	234309	141391
lus-miR156b	166353	137773	233725	141390
atr-miR156c	166349	137772	233724	141177
ahy-miR156b-5p	166348	137768	233724	141177
aly-miR157a-5p	166347	137767	233724	141175
han-miR156c	166346	137766	233724	141175
gma-miR156c	166344	137766	233723	141175
nta-miR156g	166306	137730	233664	141174
bcy-miR156	166302	137730	233664	141139
ppe-miR156i	166301	137727	233657	141135
cpa-miR156e	166291	137702	233630	141135
stu-miR156b	166286	137701	233630	141115
sly-miR156b	165951	137455	233172	141114
bra-miR158-5p	165129	124496	129341	140824

SUPPLEMENTARY TABLE S3

Sequence and function of mature and complementary strands

miRNA ID	Sequence	Biological Process
bna-miR408b-3p	AUGCACUGCCUCUUCCCUGGC	response to senescence, low copper and other abiotic stress
bna-miR408b-5p	ACAGGGAACAAGCAGAGCAUG	
bna-miR165b-3p	UCGGACCAGGCUUCAUCCCCC	adaxial/abaxial fate of leaf tissues, xylem differentiation in root stele tissues
bna-miR165b-5p	GGAAUGUUGUCUGGAUCGAGG	
bna-miR160a-5p	UGCCUGGCUCCCUGUAUGCCA	seed germination and post-germination, seedling development, symbiotic Nodule development in Soybean
bna-miR160a-3p	GCGUAUGAGGAGCCAUGCAUA	
bna-miR398-3p	UGUGUUCUCAGGUCACCCCUG	response to senescence, oxidative stress, low copper, other biotic and abiotic stress
bna-miR398-5p	GGGUCGACAUGAGAACACAUG	

SUPPLEMENTARY TABLE S4

5’ RACE primers used in this article

Primer name	Sequence
BnaA10g01450D-1R	TTCCATCTCCTTGAGAAGCTCCGGG
BnaA10g01450D-2R	CTGTGCATTCACGAGGACAGTTGTTGC
BnaC05g01480D -1R	CCATCTCCTTGAGAAGCTCCGGGAAG
BnaC05g01480D -2R	GTGCATTCACGAGGACAGTTGTTGCC
BnaC08g45940D -1R	CTCCATCTCCTTCTGAAGCTCCGGG
BnaC08g45940D -2R	TCACAAACTGTGCATTCGCGAGGACA

SUPPLEMENTARY TABLE S5

Stem-loop qRT-PCR and qRT-PCR primers used in this article

Primer name	Sequence
bna-408a-RT	CTCAACTGGTGTCGTGGAGTCCGGCAATTCAGTTGAGCCATGCTC
bna-408b-RT	CTCAACTGGTGTCGTGGAGTCCGGCAATTCAGTTGAGCATGCTCT
bna-165a-RT	CTCAACTGGTGTCGTGGAGTCCGGCAATTCAGTTGAGCCTCGATC
bna-165b-RT	CTCAACTGGTGTCGTGGAGTCCGGCAATTCAGTTGAGCCTCGATC
bna-482a-RT	CTCAACTGGTGTCGTGGAGTCCGGCAATTCAGTTGAGCTTCTTGC
bna-160a-RT	CTCAACTGGTGTCGTGGAGTCCGGCAATTCAGTTGAGTATGCATG
bna-398-RT	CTCAACTGGTGTCGTGGAGTCCGGCAATTCAGTTGAGCATGTGTT
bna-827a-RT	CTCAACTGGTGTCGTGGAGTCCGGCAATTCAGTTGAGTGTTTGTT
bna-novel-1-RT	CTCAACTGGTGTCGTGGAGTCCGGCAATTCAGTTGAGTGGAGTCA
bna-novel-2-RT	CTCAACTGGTGTCGTGGAGTCCGGCAATTCAGTTGAGCCATCATT
bna-408a-FP	ACACTCCAGCTGGGCAGGGAACAA
bna-408b-FP	ACACTCCAGCTGGGACAGGGAACA
bna-165a-FP	ACACTCCAGCTGGGGAATGTTGTC
bna-165b-FP	ACACTCCAGCTGGGGGAATGTTGT
bna-482a-FP	ACACTCCAGCTGGGAGATGGGTGG
bna-160a-FP	ACACTCCAGCTGGGGCGTATGAGG
bna-398-FP	ACACTCCAGCTGGGGGGTCGACAT
bna-827a-FP	ACACTCCAGCTGGGTTAGATGACC
bna-novel-1-FP	ACACTCCAGCTGGGAGAGTTGATC
bna-novel-2-FP	ACACTCCAGCTGGGAGACTGGAGT
Universal primer	AACTGGTGTCGTGGAG
BnaA06g40560D-RT-L	GAGGAAGGAGCAGCTGTGGACTTGC
BnaC04g00470D-RT-L	GTCCGAATAATCCACCATCCA
BnaC09g28020D-RT-L	TCCTCATCTATGATTATGCTCCC
BnaC01g28580D-RT-L	CGAGGTTCCACTGAGAAGGTGGAAC
BnaA09g32040D-RT-L	TGAAAGCTTGAACAGTCCTC
BnaC08g22860D-RT-L	GTGGGACTTCAGATACTACAACAG
BnaC08g45940D-RT-L	GATATCATTAACCTGCGGACAC
BnaA09g51130D-RT-L	TTTGGCTTCTCCTCCATCAC
BnaA10g01450D-RT-L	GGACACGATGTTTGATCCGA

BnaC05g01480D-RT-L	GCACTGTTTGATGGTTGCTC
BnaC07g31010D-RT-L	TCTACCTTCACAACCACATACAC
BnaC08g08850D-RT-L	CGCAGATTCGCTGTATGTGG
BnaC04g33280D-RT-L	CACTTATCCTCCATTGCTAACGAC
BnaC04g08980D-RT-L	GAAGGGAGAAAGCTCACCTG
BnaA06g40560D-RT-R	TCCAGTATCGTCGAGAGCCTTGGCT
BnaC04g00470D-RT-R	CTCCACTGCCAATCTGTTCTG
BnaC09g28020D-RT-R	GTAAGCAATTCCCTTAGCGA
BnaC01g28580D-RT-R	GAACGGTGGACCGAAGATCTCATCC
BnaA09g32040D-RT-R	ATCAGAAACACCAGTCTCCTC
BnaC08g22860D-RT-R	AAAGCATCAGAGACCTCATGGA
BnaC08g45940D-RT-R	TATAAACACAAACCTCACGGCA
BnaA09g51130D-RT-R	TATAAACACAAACCTCACGGCA
BnaA10g01450D-RT-R	TATAAACACAAACCTCACGGCA
BnaC05g01480D-RT-R	TATAAACACAAACCTCACGGCA
BnaC07g31010D-RT-R	TTTGCTTCTGTACCCATTAGCTG
BnaC08g08850D-RT-R	AAACCATACGGTTCTGCATCTC
BnaC04g33280D-RT-R	CTTACCTACTGCTTCCTGATGAG
BnaC04g08980D-RT-R	ACATGATGGTGGTCTTTGGT
BnActin-L	CTATCCTCCGTCTCGATCTCGC
BnActin-R	CTTAGCCGTCTCCAGCTCTTGC
