# Supplementary material for: Differential expression of miRNAs and their targets in wax-deficient rapeseed
Source: Sci Rep. 2019 Aug 21;9:12201. doi: 10.1038/s41598-019-48439-z (PMC6704058; doi:10.1038/s41598-019-48439-z)
Supplement: Supplementary file 2 — Supplementary figures [file 41598_2019_48439_MOESM2_ESM.pdf]

Supplementary Figures

Title
Differential expression of miRNAs and their targets in wax-deficient rapeseed

Tingting Liu[1], Jingquan Tang[1], Li Chen[1], Jiayue Zeng[1], Jing Wen[1], Bin Yi[1], Chaozhi Ma[1], Jinxing Tu[1], Tingdong Fu[1], Jinxiong Shen[1]*

[1] National Key Laboratory of Crop Genetic Improvement, National Center of Rapeseed Improvement, Huazhong Agricultural University, Wuhan, Hubei, China

*Corresponding author: Dr. Jinxiong Shen; jxshen@mail.hzau.edu.cn

Figure S1.

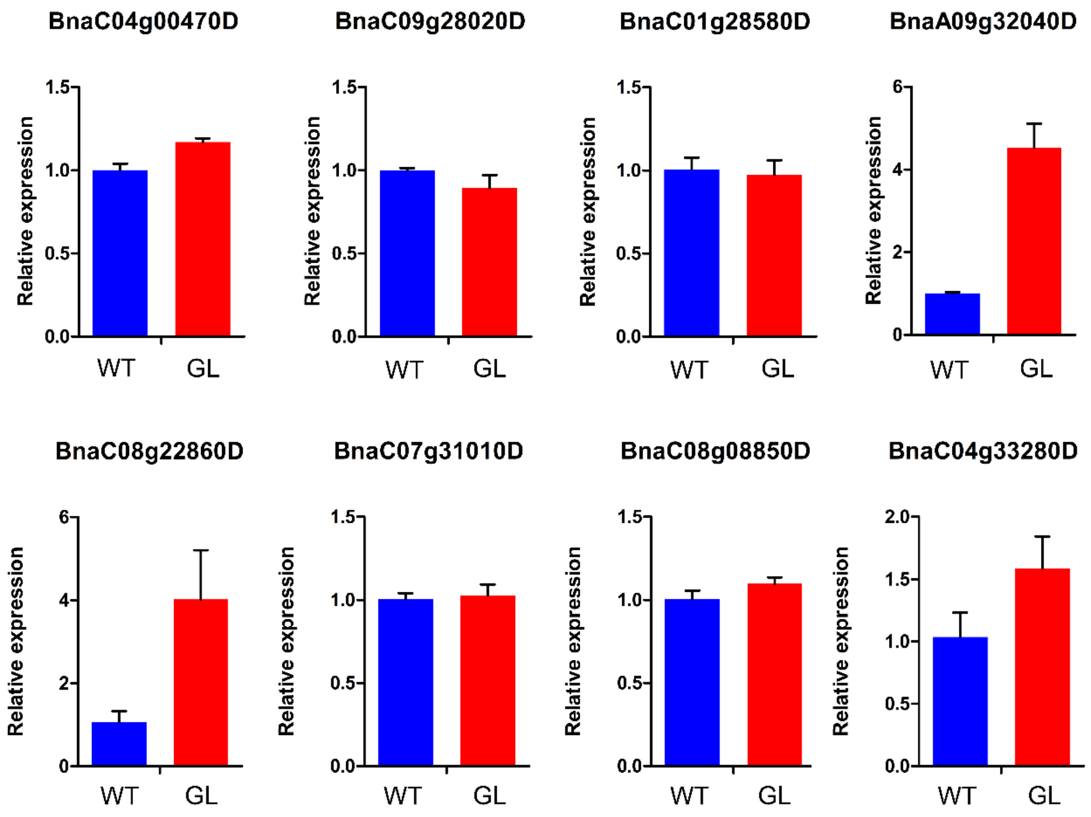

Relative expression level change of the rest of eight predicted target genes verified by qRT-PCR in wild-type and mutant plants. The bars indicate SE of the mean.

Figure S2.

A

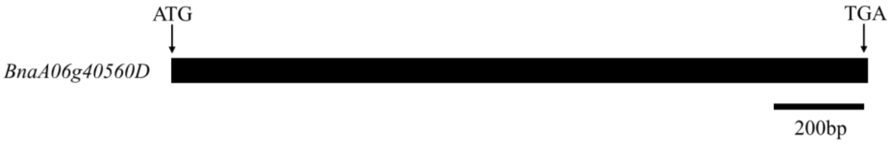

B

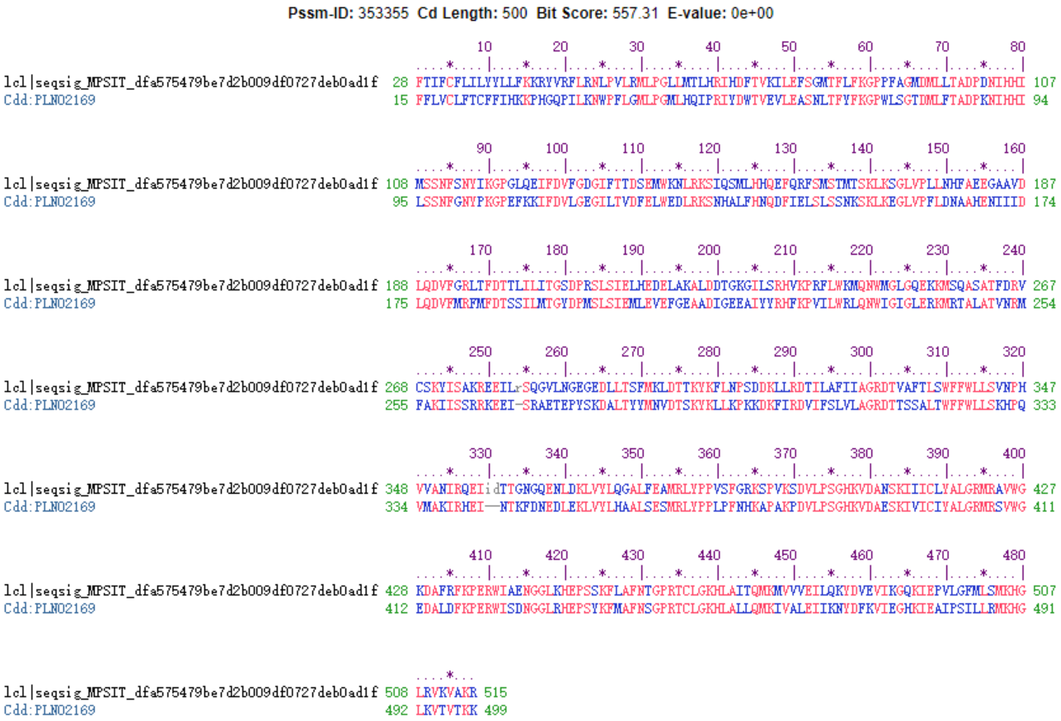

Structure analysis and function prediction of *BnaA06g40560D* gene. A. The CDS of *BnaA06g40560D* (1557bp) contains only one exon. B. The protein sequence of *BnaA06g40560D* contains the conserved domain of P450 superfamily.
